# The Population Structure of *Acinetobacter baumannii:* Expanding Multiresistant Clones from an Ancestral Susceptible Genetic Pool

**DOI:** 10.1371/journal.pone.0010034

**Published:** 2010-04-07

**Authors:** Laure Diancourt, Virginie Passet, Alexandr Nemec, Lenie Dijkshoorn, Sylvain Brisse

**Affiliations:** 1 Institut Pasteur, Genotyping of Pathogens and Public Health, Paris, France; 2 Laboratory of Bacterial Genetics, National Institute of Public Health, Prague, Czech Republic; 3 Department of Infectious Diseases, Leiden University Medical Center, Leiden, The Netherlands; University of Hyderabad, India

## Abstract

Outbreaks of hospital infections caused by multidrug resistant *Acinetobacter baumannii* strains are of increasing concern worldwide. Although it has been reported that particular outbreak strains are geographically widespread, little is known about the diversity and phylogenetic relatedness of *A. baumannii* clonal groups. Sequencing of internal portions of seven housekeeping genes (total 2,976 nt) was performed in 154 *A. baumannii* strains covering the breadth of known diversity and including representatives of previously recognized international clones, and in 19 representatives of other *Acinetobacter* species. Restricted amounts of diversity and a star-like phylogeny reveal that *A*. *baumannii* is a genetically compact species that suffered a severe bottleneck in the recent past, possibly linked to a restricted ecological niche. *A. baumannii* is neatly demarcated from its closest relative (genomic species 13TU) and other *Acinetobacter* species. Multilocus sequence typing analysis demonstrated that the previously recognized international clones I to III correspond to three clonal complexes, each made of a central, predominant genotype and few single locus variants, a hallmark of recent clonal expansion. Whereas antimicrobial resistance was almost universal among isolates of these and a novel international clone (ST15), isolates of the other genotypes were mostly susceptible. This dichotomy indicates that antimicrobial resistance is a major selective advantage that drives the ongoing rapid clonal expansion of these highly problematic agents of nosocomial infections.

## Introduction

Bacteria belonging to the species *Acinetobacter baumannii* are among the most problematic nosocomial pathogens. These organisms are notorious for their ability to colonize and infect severely ill patients in hospitals. *A. baumannii* infections are often associated with epidemic spread, and outbreak strains are frequently multidrug resistant (MDR). A most concerning development is the increasing occurrence of strains resistant to carbapenems or even to last resource antimicrobial agents including colistin or the new antibiotic tigecycline [1]–[4].

Strain typing by a variety of techniques [5], [6] has shown genotypic diversity within *A. baumannii*. Application of various methods has led to the recognition that a limited number of widespread clones are responsible for hospital outbreaks in many countries. Comparisons based on cell envelope protein profiling, ribotyping and AFLP genomic fingerprinting of epidemic and non-epidemic *A. baumannii* strains from geographically distinct European hospitals first delineated two major groups of epidemic strains, which were named European clones I and II [7]. A third pan-European outbreak clone (clone III) was subsequently distinguished based on ribotyping and AFLP [8]. The three ‘European’ clones should now more appropriately be called ‘international clones’, as they were associated with infection and epidemic spread not only in Europe, but in other parts of the world as well [9]–[19]. Multidrug resistance is often associated with isolates that belong to these international clones [7], [11], [20].

Despite the widely accepted idea that a few genotypic groups are responsible for a large proportion of the burden of *A. baumannii* infections, the genetic distinctness of clones among themselves and from other genotypes remains to be established. Fingerprinting methods provide limited phylogenetic information, results are not transportable between laboratories, and protocols and thresholds used for clone delineation may differ across studies [7], [11], [12]. In addition, genetic variation observed within clones raises the possibility that these clones harbor subtypes with distinctive temporal and geographical distributions. A standard definition of clonal relationships is needed for global epidemiological understanding and as a foundation for studying the relationships between genotype and phenotype of *A. baumannii* isolates, such as epidemic potential. Multilocus sequence typing (MLST) is the current standard for investigating the population structure of bacterial species [21]–[23]. MLST has a high potential to discriminate strains within *A. baumannii* [15], [24], [25], but has not been applied to assess the genetic structure of this species in general and of the international clones in particular.

Although *A. baumannii* is clinically the most important *Acinetobacter* species, the closely related genomic species (gen. sp.) 3 and 13TU have also been associated with nosocomial infections and outbreaks [3], [5], [26]–[28]. These three species and the environmental species *A. calcoaceticus* are genotypically closely related and phenotypically difficult to distinguish [29]–[31]. Therefore, they are sometimes referred to collectively as the *A. calcoaceticus* - *A. baumannii* (*Acb*) complex. The existence of a real phylogenetic demarcation between these closely related species is not firmly established. Multilocus sequence analysis (MLSA) of large collections of isolates belonging to closely related species has been proposed as a powerful approach to address the existence of species and to delineate their borders [32], [33].

The aims of the present study were to determine the genetic structure and diversity of *A. baumannii*, with a particular focus on the previously described international clones, and to compare antimicrobial resistance in these clones and other *A. baumannii* isolates. In addition, we determined the phylogenetic relationships and genetic distinctness of *A. baumannii* with respect to its closely related species. A set of well-described strains, mostly from clinical origin, many of which have been used in previous studies, was used.

## Results

### *A. baumannii* is well demarcated from other *Acinetobacter* species

**Figure 1:**
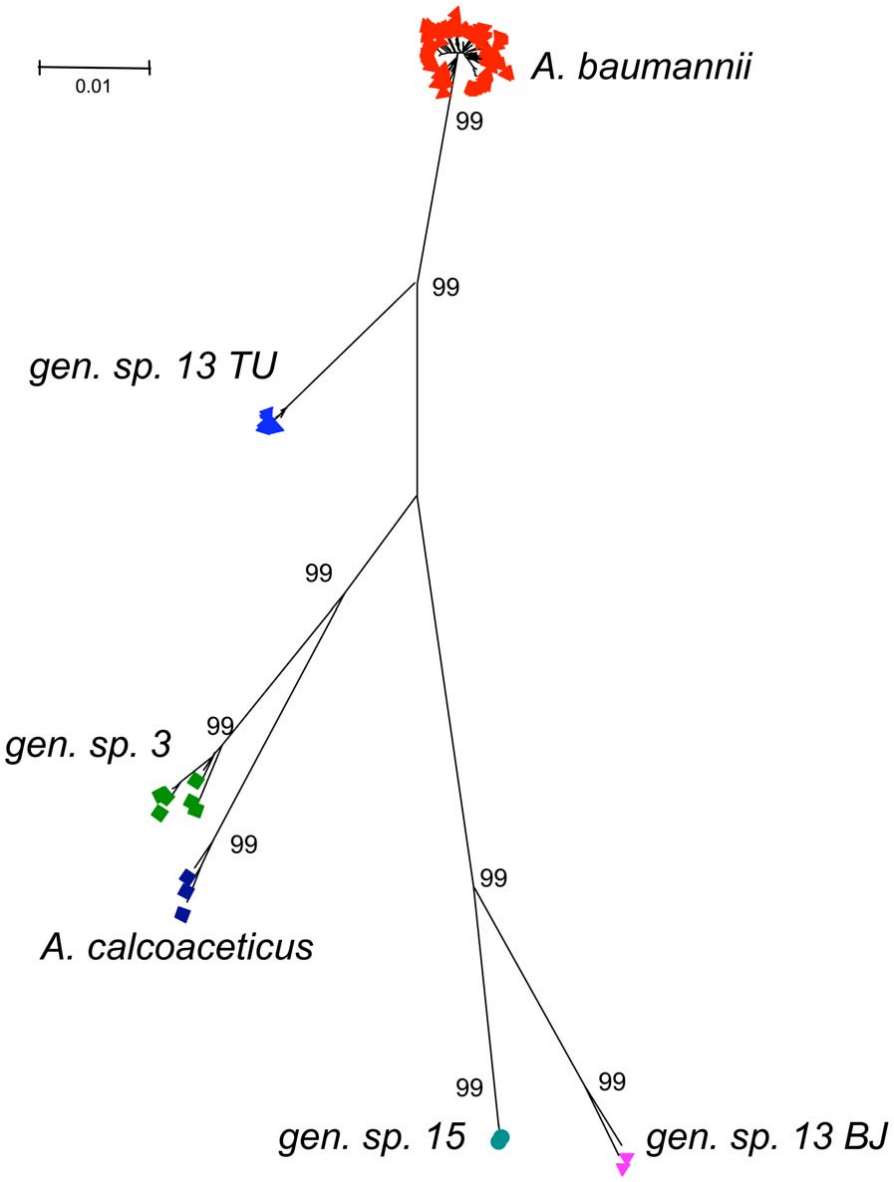
Phylogenetic analysis of 173 *Acinetobacter* strains. Concatenated sequences of seven protein-coding genes (2,976 nt in total) were compared using the neighbor-joining method and based on a Jukes-Cantor distance matrix. Bootstrap values obtained after 1,000 replicates are given at the nodes. The 154 *A. baumannii* strains clearly grouped into a compact cluster. Each of the four species of the *A. calcaoceticus-baumannii* complex was clearly distinct.

To determine the phylogenetic relationships and demarcation of *A. baumannii* from closely related species, the 154 *A. baumannii* strains were compared to the three other species of the *Acb* complex and to gen. sp. 13BJ and 15BJ. Based on the alignment of the 2,976 nucleotides of the seven genes, a total of 589 (19.8%) polymorphic sites were found. No insertion or deletion event was observed. Phylogenetic analysis of the concatenated sequences (**Figure 1**) revealed the very neat demarcation of the four species of the *Acb* complex, each forming a compact cluster separated from others by a large phylogenetic genetic distance. All species clusters had nearly maximal bootstrap support (99%), and the ratio of divergence among species to the diversity within-species (demarcation parameter [34], **Table 1**) was high for all pairwise comparisons (e.g., range 7.9 to 18 within the *Acb* complex). The phylogeny indicated that *A. baumannii* was strongly associated with gen. sp. 13TU, while gen. sp. 3 was associated with *A. calcoaceticus* (99% bootstrap support in both cases).

**Table 1:** Sequence divergence within and between *Acinetobacter* sp.

	Mean % divergence within species (a)	Mean % divergence between species (b)	Ratio (between/within) (c)
*A. baumannii* vs *A. calcoaceticus*	0.35+/−0.009; 0.83+/−0.26	9.66+/− 0.581	16.32
*A. baumannii* vs gen. sp. 3	0.35+/−0.009; 0.73+/−0.17	8.88+/−0.369	16.44
*A. baumannii* vs gen. sp. 13TU	0.35+/−0.009; 0.16+/−0.029	4.65+/−0.18	18.24
*A. baumannii* vs gen. sp. 15BJ	0.35+/−0.009; 0.034+/−0.017	11.60+/−0.8	60.42
*A. baumannii* vs gen. sp. 13BJ	0.35+/−0.009; 1.5+/−0.78	12.25+/−0.845	13.24
gen. sp. 3 vs *A. calcoaceticus*	0.73+/−0.17; 0.83+/−0.26	6.17+/−1.67	7.91
gen. sp. 13TU vs sp 3	0.16+/−0.029; 0.73+/−0.17	8.07+/−1.42	18.13
gen. sp. 13TU vs *A. calcoaceticus*	0.16+/−0.029; 0.83+/−0.26	8.83+/−2.24	17.84

(a) Mean +/− standard error for pairwise divergence within each of the species shown in order of appearance in the comparison column.

(b) Mean +/− standard error for pairwise divergence between the species, based on all pairwise comparisons of strains from different species.

(c) *k* parameter: Ratio of the between-species divergence to the average of the within-species divergence levels (Palys et al. 1997).

Comparison of the phylogenies obtained using each gene individually showed strong congruence among the seven genes (**Figure S1**). However, some discrepancies were observed. For example, as opposed to the concatenate and to five individual genes, *rplB* did not associate *A. baumannii* strongly with gen. sp. 13TU. On the contrary, in the *rplB* phylogeny, all isolates of species 13TU were associated in a short, strongly supported branch with species 3 and with *A. calcoaceticus* (**Figure S1**). This observation can be attributed to the horizontal transfer of the *rplB* gene from a donor related to *A. calcoaceticus* and gen. sp. 3 into an ancestral strain of gen. sp. 13TU. Gene *rpoB* showed an intermediate situation for the position of gen. sp. 13TU, which was neither strongly associated with *A. baumannii* nor with *A. calcoaceticus* and gen. sp. 3, consistent with previous findings [35]. Interestingly for the purpose of strain identification, no single isolate was placed in a species cluster distinct from the one it belongs to based on concatenated sequences, showing that replacement of genomic sequences by homologous DNA from other species is not frequent.

### Restricted nucleotide diversity and lack of phylogenetic structure within *A. baumannii*

The proportion of variable sites observed among the 154 *A. baumannii* strains varied from 2% (*pyrG*) to 4.8% (*recA*) (**Table 2**). Considering the seven genes together, there were 95 variable sites, including 55 parsimony-informative ones. Non-synonymous substitutions were rare compared to synonymous substitutions (**Table 2**), indicating selection against amino acid changes, consistent with the expectation of purifying selection acting on housekeeping genes. The nucleotide diversity (π, average number of nucleotide differences per site between two randomly-selected strains) ranged from 0.2% (*fusA*) to 0.76% (*recA*) on the entire population, and from 0.26% (*pyrG*) to 0.85% (*recA*) based on unique STs only (excluding a bias towards low diversity due to the incorporation of multiple isolates of the major clones and the seven outbreaks). Hence, the level of divergence of the core genome within *A. baumannii* is strikingly lower than between *A. baumannii* and its closest species, 13TU (4.6% on average).

**Table 2:** Polymorphism among 154 strains of *A. baumannii*.

Gene	Size (bp)	No. of alleles	No. of polymorphic sites (non-synonymous sites)	dN	dS	dN/dS	π (%)	π on STs (%)
*cpn60*	405	13	12 (0)	0	0.01981	0.000	0.448	0.38
*fusA*	633	17	16 (3)	0.00042	0.00768	0.055	0.204	0.27
*gltA*	483	19	14(0)	0	0.01137	0.000	0.253	0.34
*pyrG*	297	7	6 (0)	0	0.00942	0.000	0.219	0.26
*recA*	372	18	18 (0)	0	0.03262	0.000	0.756	0.85
*rplB*	330	9	8 (0)	0	0.01057	0.000	0.27	0.27
*rpoB*	456	16	16 (2)	0.00011	0.01526	0.0072	0.375	0.39
Concatenate	2,976	58	89 (5)	0.00011	0.01487	0.0074	0.35	0.385

dN: non-synonymous substitutions per non-synonymous site.

dS: synonymous substitutions per synonymous site.

π: average number of nucleotide differences per site between two randomly-selected strains. The value is given for 100 sites.

The existence of a phylogenetic pattern within *A. baumannii* was investigated by neighbor-joining analysis of the concatenated sequences of the seven genes (**Fig. S2**). There was no evidence of phylogenetic structuring, as no subsets of strains were clearly separated from others. Instead, most sequences appeared roughly equidistant, with the exception of a few tight terminal clusters that correspond to clonal complexes (see below). There was no evidence that these clonal complexes had a common evolutionary origin.

### Genotypic diversity within *A. baumannii* and identification of international clones

**Figure 2:**
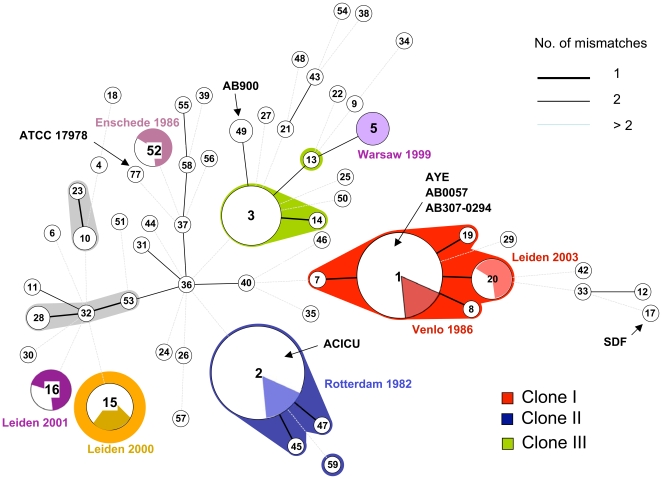
Minimum spanning tree analysis of 154 strains of *A. baumannii*. The number of allelic mismatches among MLST profiles was used as distance. Each circle corresponds to one sequence type (ST), with its number indicated inside. Circle size increases logarithmically with the number of isolates that had this ST, from one (smallest circles) to 33 (ST2). Colored or grey zones that surround some groups of circles indicate that these profiles belong to the same clonal complex (CC), meaning that they have a single allelic mismatch with at least one other member of the group. Multiresistant clones CC1, CC2, CC3 and ST15 are colored. The colored pie chart sections inside circles indicate the proportion of strains that were part of one of the seven outbreak sets, the location and year of which is indicated besides the corresponding circle, in the same color. Seven genome reference strains are indicated in bold. Note that the inferred relationships displayed among STs differing by more than one allelic mismatch should not be considered as reliable, as many alternative links with the same number of mismatches often exist.

The *A. baumannii* strains were grouped by MLST into 59 distinct sequence types (ST). Forty-seven STs corresponded to a single isolate, whereas three STs comprised 15 strains or more (ST1, n = 24, ST2, n = 33 and ST3, n = 15). These three STs, comprising 46% of the strains altogether, were composed of strains previously identified as international clone I, II and III, respectively (**Table 3**). Relationships among genotypes were disclosed using the MStree method (**Figure 2**). Only five clonal complexes (CC) were found, three of which corresponded to international clones I–III. CC1 comprised all strains previously determined to belong to clone I, including its reference strain RUH875. CC1 was composed of ST1, ST7, ST8, ST19 and ST20. Whereas the four latter STs differed from ST1 by a single allelic mismatch, they differed among themselves by two mismatches, indicating that ST1 is the probable founder genotype of CC1, from which the other STs evolved by a single allelic change. The fact that ST1 was also, by far, the most frequent among these five STs, indicates that ST1 experienced a clonal expansion.

**Table 3:** Strains used in this study and their characteristics.

Species/strain code(s)	Clone by MLST	ST	Allelic profile	Clone or cluster by AFLP	AFLP cluster no (80%)	AFLP cluster no. (90%)	No. of resistancies*	MDR **	Outbreak/cross infection ***	City, country, year of isolation	Source	Reference for strain source
***A. baumannii*** ****												
LUH 3783 ( = NIPH 10)	CC1	1	1-1-1-1-5-1-1	I	34	66	8	+	Yes	Prague, CZ, 1991	Blood	Nemec et al JMM 2004a, Nemec et al JAC 2007
LUH 4576 ( = NIPH 56)	CC1	1	1-1-1-1-5-1-1	I	34	67	2	-	Unknown	Prague, CZ, 1992	Burn	Nemec et al JMM 2004a, Nemec et al JAC 2007
LUH 4624 ( = NIPH 470)	CC1	1	1-1-1-1-5-1-1	I	34	64	6	+	Unknown	C. Budejovice, CZ, 1997	Bronchus	Nemec et al JMM 2004a, Nemec et al JAC 2007
LUH 4640 ( = NIPH 321)	CC1	1	1-1-1-1-5-1-1	I	34	70	7	+	Unknown	Tábor, CZ, 1994	Urine	Nemec et al JMM 2004a, Nemec et al JAC 2007
LUH 6015 ( = 11A352)	CC1	1	1-1-1-1-5-1-1	I	34	72	5	+	Yes	Rome, IT, 1998	Blood	van Dessel et al Res Microbiol 2003
LUH 6224	CC1	1	1-1-1-1-5-1-1	I	34	70	8	+	Yes	Sydney, AU, 1995	Blood	Valenzuela J Clin Microbiol 2007;45:453
LUH 7140 ( = A789)	CC1	1	1-1-1-1-5-1-1	I	34	64	6	+	Yes	London, UK, 2000	Sputum	
LUH 8592	CC1	1	1-1-1-1-5-1-1	I	34	63	7	+	No	Sofia, BG, 2001	Urine	Dobrevski et al 2006
LUH 9668	CC1	1	1-1-1-1-5-1-1	I	34	71	6	+	Unknown	Dublin, IE, 2003	Wound (horse)	Abbott et al JAC 2005
RUH 0875	CC1	1	1-1-1-1-5-1-1	I	34	69	6	+	Yes	Dordrecht, NL, 1984	Urine	Dijkshoorn et al JCM 1996, Janssen et al IJSB 1997
RUH 2037	CC1	1	1-1-1-1-5-1-1	I	34	68	7	+	Yes (Outbreak 1, n = 4)	Venlo, NL, 1986	Sputum	Crombach et al ICM 1989, Dijkshoorn et al JCM 1992
RUH 3238 ( = GNU 1084)	CC1	1	1-1-1-1-5-1-1	I	34	70	5	+	Yes	Sheffield, UK, 1987	Burn	Dijkshoorn et al JCM 1996
RUH 3239 ( = GNU 1083)	CC1	1	1-1-1-1-5-1-1	I	34	68	6	+	Yes	London, UK, 1985-88	Urine	Dijkshoorn et al JCM 1996
RUH 3242 ( = GNU 1082)	CC1	1	1-1-1-1-5-1-1	I	34	71	6	+	Yes	Basildon, UK, 1989	Burn	Dijkshoorn et al JCM 1996
RUH 3247 ( = GNU 1078)	CC1	1	1-1-1-1-5-1-1	I	34	71	7	+	Yes	Leuven, BE, 1990	Rectum	Dijkshoorn et al JCM 1996
RUH 3282 ( = GNU 1079)	CC1	1	1-1-1-1-5-1-1	I	34	71	8	+	Yes	Salford, UK, 1990	Tracheostomy	Dijkshoorn et al JCM 1996
LUH 6014 ( = 11A221)	CC1	1	1-1-1-1-5-1-1	I	34	72	7	+	Yes	Rome, IT, 1998	Blood	van Dessel et al Res Microbiol 2003
LUH 6050 ( = 36C058)	CC1	1	1-1-1-1-5-1-1	I	34	62	7	+	Yes	Pretoria, ZA	Respiratory tract	van Dessel et al Res Microbiol 2003
LUH 6013 ( = 11A018)	CC1	1	1-1-1-1-5-1-1	I	35	74	6	+	Yes	Rome, IT, 1997	Blood	van Dessel et al Res Microbiol 2003
LUH 5881 ( = 17C078)	CC1	1	1-1-1-1-5-1-1	I	34	73	7	+	Yes	Madrid, ES, 1998	Respiratory tract	van Dessel et al Res Microbiol 2003
LUH 6125 ( = 14C052)	CC1	1	1-1-1-1-5-1-1	I	34	64	7	+	Yes	Krakow, PL, 1998	Respiratory tract	van Dessel et al Res Microbiol 2003
AYE	CC1	1	1-1-1-1-5-1-1	nd	Nd	nd	nd	+ *****	Yes *****	Kremlin-Bicetre, F, 2001	Urine	Poirel et al JCM 2003
AB0057	CC1	1	1-1-1-1-5-1-1	nd	Nd	nd	nd	+ *****	nd	Washington D.C., USA, 2004	Blood	Adams et al 2008
AB307-0294	CC1	1	1-1-1-1-5-1-1	nd	Nd	nd	nd	- *****	nd	Buffalo, NY, 1994	Blood	Adams et al 2008
LUH 3782 ( = NIPH 7)	CC1	7	1-1-1-2-5-1-1	I	34	66	7	+	Unknown	Prague, CZ, 1991	Burn	Nemec et al JMM 2004a, Nemec et al JAC 2007
RUH 0510	CC1	8	1-1-1-1-1-1-1	I	34	71	5	+	Yes	Nijmegen, NL, 1984	Bronchus	Janssen et al IJSB 1997, Dijkshoorn et al JCM 1996
LUH 8605	CC1	19	1-2-1-1-5-1-1	I	36	75	7	+	No	Sofia, BG, 2002	Wound	Dobrewski et al CMI 2006
LUH 8723	CC1	20	3-1-1-1-5-1-1	I	34	65	8	+	Yes (Outbreak 6, n = 5)	Leiden, NL, 2003	Wound	
A1755	CC2	2	2-2-2-2-2-2-2	II	27	46	nd	nd	No	Chelmsford, UK, 2000	Wound	
LUH 3788 ( = NIPH 24)	CC2	2	2-2-2-2-2-2-2	II	27	40	6	+	Unknown	Prague, CZ, 1991	Urine	Nemec et al JMM 2004a, Nemec et al JAC 2007
LUH 4629 ( = NIPH 657)	CC2	2	2-2-2-2-2-2-2	II	27	40	7	+	Yes	Prague, CZ, 1996	Tracheostomy	Nemec et al JMM 2004a, Nemec et al JAC 2007
LUH 5682	CC2	2	2-2-2-2-2-2-2	II	27	41	5	+	Unknown	Utrecht, NL, 1993	Catheter (horse)	
LUH 6024 ( = 16A502)	CC2	2	2-2-2-2-2-2-2	II	27	45	7	+	Yes	Sevilla, ES, 1998	Blood	van Dessel et al Res Microbiol 2003
LUH 8065	CC2	2	2-2-2-2-2-2-2	II	27	47	8	+	Yes	Amsterdam, NL, 2001	Hospital env.	van den Broek et al CMI 2006
LUH 8488	CC2	2	2-2-2-2-2-2-2	II	27	46	7	+	Unknown	Leeuwarden, NL, 2003	Wound	
LUH 9233 ( = NIPH 1945)	CC2	2	2-2-2-2-2-2-2	II	27	43	7	+	Unknown	Prague, CZ, 2003	Sputum	Nemec et al JAC 2007
RUH 0134	CC2	2	2-2-2-2-2-2-2	II	27	40	5	+	Yes (Outbreak 2, n = 6)	Rotterdam, NL, 1982	Urine	Janssen et al IJSB 1997
RUH 3240 ( = GNU 1086)	CC2	2	2-2-2-2-2-2-2	II	27	44	4	+	Yes	Newcastle, UK, 1989	Respiratory tract	Dijkshoorn et al JCM 1996
RUH 3245 ( = GNU 1080)	CC2	2	2-2-2-2-2-2-2	II	27	45	3	+	Yes	Salisbury, UK, 1989	Urine	Dijkshoorn et al JCM 1996
RUH 3422 ( = PGS 189)	CC2	2	2-2-2-2-2-2-2	II	27	40	1	-	No	Odense, DK, 1984	Crural ulcer	Dijkshoorn et al JCM 1996
LUH 6025 ( = 16A528)	CC2	2	2-2-2-2-2-2-2	II	27	45	7	+	Yes	Sevilla, ES, 1998	Blood	van Dessel et al Res Microbiol 2003
LUH 6045 ( = 18C144)	CC2	2	2-2-2-2-2-2-2	II	27	44	9	+	Yes	Barcelona, ES, 1997	Sputum	van Dessel et al Res Microbiol 2003
LUH 6051 ( = 36D042)	CC2	2	2-2-2-2-2-2-2	II	27	45	4	+	Yes	Pretoria, ZA	Wound	van Dessel et al Res Microbiol 2003
LUH 5868 ( = 06A102)	CC2	2	2-2-2-2-2-2-2	II	27	45	9	+	Yes	Lille, FR, 1997	Blood	van Dessel et al Res Microbiol 2003
LUH 6021 ( = 14C003)	CC2	2	2-2-2-2-2-2-2	II	27	42	8	+	Yes	Krakow, PL, 1998	Sputum	van Dessel et al Res Microbiol 2003
LUH 7154 ( = A1850)	CC2	2	2-2-2-2-2-2-2	II	28	53	5	+	No	Berkshire, UK, 2000	Urine	Spence et al JCM 2004
LUH 8143	CC2	2	2-2-2-2-2-2-2	II	28	52	7	+	Yes	Singapore, SG, 1997	Sputum	
LUH 8533	CC2	2	2-2-2-2-2-2-2	II	28	51	7	+	Unknown	London, UK,	Urine	
RUH 3381 ( = GNU 666)	CC2	2	2-2-2-2-2-2-2	II	28	50	3	+	Unknown	Cork, IE, 1989	Sputum	
LUH 5089	CC2	2	2-2-2-2-2-2-2	II	27	49	6	+	Unknown	Warsaw, PL, before 1999	Ascites	
LUH 6231	CC2	2	2-2-2-2-2-2-2	II	27	48	9	+	Yes	Sydney, AU, 1999	Hip	Valenzuela J Clin Microbiol 2007;45:453
LUH 6038 ( = 18A350)	CC2	2	2-2-2-2-2-2-2	II	29	54	9	+	Yes	Barcelona, ES, 1998	Blood	van Dessel et al Res Microbiol 2003
LUH 6034 ( = 17C003)	CC2	2	2-2-2-2-2-2-2	II	29	54	10	+	Yes	Madrid, ES, 1997	Sputum	van Dessel et al Res Microbiol 2003
LUH 6126 ( = 15A250)	CC2	2	2-2-2-2-2-2-2	II	27	45	7	+	Yes	Coimbra, PT, 1998	Blood	van Dessel et al Res Microbiol 2003
ACICU	CC2	2	2-2-2-2-2-2-2	II	Nd	nd	nd	+ *****	nd	Rome, IT, 2005	Cerebrospinal fluid	Iacono et al AAC 2008
LUH 6011 ( = 09A242)	CC2	45	2-6-2-2-2-2-2	II	27	44	7	+	Yes	Athens, GR, 1997	Blood	van Dessel et al Res Microbiol 2003
LUH 7855 ( = NIPH 1362)	CC2	47	2-13-2-2-2-2-2	II	40	79	5	+	Yes	Prague, CZ, 2000	Tracheal aspirate	Nemec et al JMM 2004a, Nemec et al JAC 2007
LUH 5875 ( = 12A133)	CC3	3	3-3-2-2-3-1-3	III	32	58	8	+	Yes	Utrecht, NL, 1997	Blood	van Dessel et al Res Microbiol 2003
LUH 6009 ( = 04C048)	CC3	3	3-3-2-2-3-1-3	III	32	58	8	+	Yes	Paris, FR, 1997	Sputum	van Dessel et al Res Microbiol 2003
LUH 6012 ( = 10C070)	CC3	3	3-3-2-2-3-1-3	III	32	58	8	+	Yes	Genoa, IT, 1998	Sputum	van Dessel et al Res Microbiol 2003
LUH 6028 ( = 16D025)	CC3	3	3-3-2-2-3-1-3	III	32	58	7	+	Yes	Sevilla, ES, 1998	Wound	van Dessel et al Res Microbiol 2003
LUH 6035 ( = 17C085)	CC3	3	3-3-2-2-3-1-3	III	32	58	8	+	Yes	Madrid, ES, 1998	Sputum	van Dessel et al Res Microbiol 2003
LUH 6037 ( = 18A155)	CC3	3	3-3-2-2-3-1-3	III	32	58	8	+	Yes	Barcelona, ES, 1997	Blood	van Dessel et al Res Microbiol 2003
LUH 6215	CC3	3	3-3-2-2-3-1-3	III	32	58	8	+	Unknown	Heerlen, NL, 2000	Skin	
LUH 8056	CC3	3	3-3-2-2-3-1-3	III	32	60	8	+	Yes	Groningen, NL, 2000	Hospital env.	van den Broek et al CMI 2006
LUH 9536	CC3	3	3-3-2-2-3-1-3	III	32	57	7	+	Yes	Gent, BE, 1993	Sputum	Huys et al RM 2005
LUH 6020 ( = 12A126)	CC3	3	3-3-2-2-3-1-3	III	32	58	7	+	Yes	Utrecht, NL, 1997	Blood	van Dessel et al Res Microbiol 2003
LUH 6030 ( = 16D083)	CC3	3	3-3-2-2-3-1-3	III	32	58	7	+	Yes	Sevilla, ES, 1997	Wound	van Dessel et al Res Microbiol 2003
LUH 6036 ( = 18A025)	CC3	3	3-3-2-2-3-1-3	III	32	58	7	+	Yes	Barcelona, ES, 1997	Blood	van Dessel et al Res Microbiol 2003
LUH 6048 ( = 18D047)	CC3	3	3-3-2-2-3-1-3	III	32	57	0	-	Yes	Barcelona, ES, 1997	Wound	van Dessel et al Res Microbiol 2003
LUH 5874 ( = 06A201)	CC3	14	3-3-2-2-3-1-7	III	32	58	6	+	Yes	Lille, FR, 1997	Blood	van Dessel et al Res Microbiol 2003
LUH 4641 ( = NIPH 335)	CC10	10	1-3-2-1-4-4-4	cluster B	25	36	6	+	Unknown	Tábor, CZ, 1994	Sputum	Nemec et al JMM 2004a, Nemec et al JAC 2007
LUH 6237	CC10	10	1-3-2-1-4-4-4	cluster B	25	35	1	-	No	Darwin, AU, 1981-91	Blood	
RUH 1316	CC10	23	1-3-10-1-4-4-4	cluster B	25	37	0	-	Unknown	Rotterdam, NL, 1964	Mink	
LUH 8406 ( = NIPH 1734)	ST15	15	6-6-8-2-3-5-4	cluster A	1	2	7	+	Unknown	M. Boleslav, CZ, 2001	Sputum	Nemec et al JMM 2004a, Nemec et al JAC 2007
LUH 6374	ST15	15	6-6-8-2-3-5-4	cluster A	1	3	5	+	Yes (Outbreak 4, n = 3)	Leiden, NL, 2000	Pharynx	van den Broek et al 2009
LUH 8102	ST15	15	6-6-8-2-3-5-4	cluster A	1	1	6	+	Yes	Tilburg, NL, 2000	Wound	van den Broek et al CMI 2006
LUH 8147	ST15	15	6-6-8-2-3-5-4	cluster A	1	5	4	+	Yes	Buenos Aires, AR, 1995	Sputum	
LUH 8850	ST15	15	6-6-8-2-3-5-4	cluster A	1	1	6	+	Unknown	Leiden, NL, 2003	Pus	
LUH 9716	ST15	15	6-6-8-2-3-5-4	cluster A	1	4	10	+	Yes	Ede, NL, 2004	Drain bowel	
RUH 2208	CC32	28	1-1-2-2-10-4-4	cluster 6	17	26	0	-	No	Malmö, SE, 1980-81	Wound	Janssen et al 1997, Dijkshoorn et al JCM 1996
RUH 3428	CC32	28	1-1-2-2-10-4-4	cluster 6	17	27	0	-	No	Malmö, SE, 1980-81	Wound	Dijkshoorn et al JCM 1996, Tjernberg & Ursing APMIS 1989
RUH 3425	CC32	32	1-1-2-2-3-4-4	cluster 6	7	14	0	-	No	Veile, DK, 1990	Urine	Dijkshoorn et al JCM 1996
RUH 2207	CC32	53	1-1-2-2-3-4-2		19	29	0	-	No	Malmö, SE, 1980-81	Sputum	Janssen et al IJSB 1997, Tjernberg & Ursing, 1989
RUH 3023^T^ ( = ATCC19606^T^)	ST52	52	3-2-2-7-9-1-5	cluster C	7	16	2	-	Unknown	Before 1949	Urine	Janssen et al IJSB 1997, Nemec et al JAC 2007
RUH 1752	ST52	52	3-2-2-7-9-1-5	cluster C	7	15	0	-	Yes (Outbreak 7, n = 3)	Enschede, NL, 1986	Bronchus	Dijkshoorn et al JCP 1991
RUH 1063 ( = NCTC 7844)	ST52	52	3-2-2-7-9-1-5	cluster C	7	15	2	-	Unknown	Before 1948	Unknown	Janssen et al IJBS 1997, Nemec et al JAC 2007
LUH 8225		4	1-3-3-2-4-1-4		15	24	0	-	No	Leiden, NL, 2002	Bronchus	van den Broek et al 2009
LUH 5703		5	4-1-2-2-4-1-5		22	32	7	+	Yes (Outbreak 3, n = 4)	Warsaw, PL, 1999	Cerebrospinal fluid	Wroblewska et al JHI 2004
A955		6	5-4-4-1-3-3-4		2	6			No	London, UK, 2000	Bronchus	
LUH 4633 ( = NIPH 190)		9	3-1-5-3-6-1-3		44	83	0	-	Unknown	Prague, CZ, 1993	Tracheostomy	Nemec et al JMM 2004a, Nemec et al JAC 2007
LUH 4718 ( = NIPH 329)		11	1-2-6-2-3-4-4		18	28	0	-	Unknown	Tábor, CZ, 1994	Tracheostomy	Nemec et al JMM 2004a, Nemec et al JAC 2007
LUH 4727 ( = NIPH 615)		12	3-5-7-1-7-2-6		5	10	0	-	Unknown	Prague, CZ, 1994	Tracheostomy	Nemec et al JMM 2004a, Nemec et al JAC 2007
LUH 5687		13	3-1-2-2-4-1-3	III	32	59	0	-	Unknown	Utrecht, NL, 1996	Throat (dog)	
LUH 6639		16	7-7-2-2-8-4-4		37	76	8	+	Yes (Outbreak 5, n = 6)	Leiden, NL, 2001	Drain tip	Bernards et al 2004, van den Broek et al 2006
SDF		17	3-29-30-1-9-1-4		Nd	nd	nd	-	no	F, <1999	Body louse	Fournier et al 2006, Vallenet et al 2008
LUH 8326		18	1-8-9-2-4-6-4		24	34	0	-	No	Leiden, NL, 2002	Wound	van den Broek et al 2009
LUH 9415		21	3-3-2-2-4-4-8		23	33	0	-	No	Leiden, NL, 2004	Sputum	van den Broek et al 2009
RUH 1093		22	3-9-3-2-4-1-9		7	13	2	-	No	Rotterdam, NL, 1985	Sputum	Janssen et al IJBS 1997, Dijkshoorn et al JCM 1996
RUH 1317		24	1-10-2-2-9-1-10		9	18	0	-	Unknown	Rotterdam, NL, 1965	Mink	
RUH 1486		25	3-3-2-4-7-2-4		4	8	0	-	No	Rotterdam, NL, 1985	Umbilicus	
RUH 1907		26	1-2-11-5-3-1-11		26	39	0	-	No	Rotterdam, NL, 1986	Bronchus	Dijkshoorn et al JCM 1996,
RUH 2180		27	3-3-12-2-9-7-4		12	21	0	-	No	Nijmegen, NL, 1987	Sputum	
RUH 3413		29	1-3-13-1-5-8-12		6	12	3	+	No	London, UK, 1981	Skin	Dijkshoorn et al JCM 1996
RUH 3423		30	1-1-2-5-3-2-3		45	84	0	-	No	Naestved, DK, 1990	Urine	Dijkshoorn et al JCM 1996
RUH 3424		31	1-2-2-2-11-1-1		16	25	2	-	No	Veile, DK, 1990	Urine	Dijkshoorn et al JCM 1996
RUH 3429		33	8-1-14-3-12-1-13		4	9	1	-	No	Malmö, SE, 1980-81	Wound	Dijkshoorn et al JCM 1996,Tjernberg & Ursing APMIS 1989
LUH 4631 ( = NIPH 60)		34	9-3-2-2-5-4-14		30	55	0	-	Unknown	Prague, CZ, 1992	Sputum	Nemec et al JMM 2004a, Nemec et al JAC 2007
LUH 4707 ( = NIPH 67)		35	1-2-2-2-3-1-2		25	38	0	-	Unknown	Prague, CZ, 1992	Tracheostomy	Nemec et al JMM 2004a, Nemec et al JAC 2007
LUH 4708 ( = NIPH 70)		36	3-2-2-2-7-1-2		38	77	0	-	Unknown	Prague, CZ, 1992	Tracheostomy	Nemec et al JMM 2004a, Nemec et al JAC 2007
LUH 4709 ( = NIPH 80)		37	3-2-15-6-6-4-5		20	30	1	-	Unknown	Prague, CZ, 1993	I. v. catheter	Nemec et al JMM 2004a, Nemec et al JAC 2007
LUH 4711 ( = NIPH 201)		38	10-4-3-2-13-1-2		31	56	0	-	Unknown	Liberec, CZ, 1992	Nose	Nemec et al JMM 2004a, Nemec et al JAC 2007
LUH 4722 ( = NIPH 410)		39	1-2-2-2-5-1-14		3	7	0	-	Unknown	Brno, CZ, 1996	Cannula	
LUH 4725 ( = NIPH 601)		40	1-1-2-2-12-1-5		43	82	0	-	Unknown	Prague, CZ, 1993	Urine	Nemec et al JMM 2004a, Nemec et al JAC 2007
LUH 5684		42	3-11-16-1-13-1-15		8	17	2	-	Unknown	Utrecht, NL, 1994	Blood (horse)	
LUH 5685		43	3-3-13-2-4-4-5		33	61	0	-	Unknown	Utrecht, NL, 1994	Nose (dog)	
LUH 5691		44	11-2-2-4-13-1-2		11	20	0	-	Unknown	Utrecht, NL, 1997	Eye (cat)	
LUH 7852 ( = NIPH 301)		46	5-12-11-2-14-9-14		39	78	7	+	Unknown	Slaný, CZ, 1994	Sputum	Nemec et al JMM 2004a
LUH 8088		48	3-14-2-2-15-4-5		10	19	0	-	No	Leiden, NL, 2002	Sputum	van den Broek et al 2009
LUH 9084		49	3-3-6-2-3-1-5		41	80	0	-	No	Leiden, NL, 2003	Urine	van den Broek et al 2009
AB900		49	3-3-6-2-3-1-5		nd	nd	nd	- *****	nd	Washington D.C., USA, 2003	Perinea	Adams et al 2008
LUH 9136		50	3-15-17-2-3-1-2		13	22	3	+	No	Leiden, NL, 2004	Sputum	van den Broek et al 2009
RUH 0414		51	3-16-6-2-16-4-2		6	11	0	-	Unknown	Leiden, NL, 1978	Auditory canal	Dijkshoorn et al JCM 1996
RUH 2209 ( = ATCC 17904)		54	12-3-18-2-17-4-5		46	85	1	-	Unknown	Before 1962	Urine	Janssen et al IJSB 1997, Tjernberg & Ursing, 1989
RUH 2688		55	13-4-2-2-6-1-16		42	81	2	-	No	Rotterdam, NL, 1987	Throat	Dijkshoorn et al JCM 1996
RUH 3410		56	3-17-7-2-18-1-2		14	23	0	-	No	London, UK, 1982	Skin	Dijkshoorn et al JCM 1996
RUH 3414		57	1-3-17-5-3-1-14		21	31	0	-	No	London, UK, 1988	Nail fold	Dijkshoorn et al JCM 1996
SB 1414		58	13-4-2-2-7-1-2		nd	nd	nd	-	Unknown	Utrecht, NL, 1997	Blood	
LUH 6049		59	3-2-19-2-5-2-5	II	27	41	6	+	Unknown	Ankara, TR, 1997	Wound	
ATCC 17978		77	3-2-2-2-3-4-28		nd	nd	nd		Unknown	1951	Meningitis	Smith et al Genes Dev 2007
***A. calcoaceticus***												
RUH 2201^T^ ( = ATCC23055^T^)		62	16-19-22-9-19-12-19		53	92	0	-	Unknown	Delft, NL, before 1911	Soil	
CIP 6632		60	14-18-20-8-19-10-17		nd	nd	nd			Unknown	Unknown	
LUH 9144		61	15-18-21-8-19-11-18		54	93	0	-	No	Leiden, NL, 2004	Urine	van den Broek et al 2009
***A.*** ** genomic sp. 3**												
RUH 2206 ( = ATCC 19004)		63	17-20-23-10-20-13-20		57	96	0	-	Unknown	Unknown	Cerebrospinal fluid	Janssen et al IJSB 1997, Tjernberg & Ursing 1989
RUH 1944		70	23-20-23-16-25-18-20		59	98	1	-	Yes	The Hague, NL, 1986	Urine	Dijkshoorn et al JCM 1993
RUH 0509		72	24-27-27-17-20-18-20		58	97	0	-	No	Nijmegen, NL, 1984	Bronchus	Dijkshoorn et al JCP 1993, Janssen et al IJSB 1997
RUH 2204		73	23-28-28-10-25-18-26		55	94	0	-	No	Malmoe, SE, 1980-81	Wound	Tjernberg & Ursing 1989, Janssen et al IJSB 1997
RUH 1163		75	17-21-23-10-20-13-27		56	95	0	-	No	Rotterdam, NL, 1985	Toe web	Janssen et al IJSB 1997
***A.*** ** genomic sp. 13TU**												
RUH 0503		68	20-24-26-14-23-16-23		50	89	2	-	No	Nijmegen, NL, 1984	Urine	Janssen et al IJSB 1997
RUH 3417		68	20-24-26-14-23-16-23		50	89	3	-	Yes	Odense, DK,	Respiratory tract	Dijkshoorn et al JCM 1993
RUH 2210 ( = ATCC 17903)		74	22-26-29-14-27-16-23		52	91	0	-	Unknown	Before 1968	Unknown	Janssen et al IJSB 1997
LUH 7715		71	20-26-26-14-26-16-25		48	87	0	-	Yes	Utrecht, NL, 2000	Sputum	van Dessel et al JHI 2002
LUH 8731		71	20-26-26-14-26-16-25		47	86	1	-	Yes	Leiden, NL, 2003	Sputum	
RUH 2624		71	20-26-26-14-26-16-25		49	88	1	-	No	Rotterdam, NL, 1987	Skin	Janssen et al IJSB 1997
RUH 2376		76	20-26-26-18-27-19-23		51	90	0	-	Unknown	Rotterdam, NL, 1987	Sputum	Janssen et al IJSB 1997
***A.*** ** genomic sp. 13BJ**												
LUH 1718 ( = SEIP 5.84)		65	18-22-24-11-21-14-21		60	99	0	-	Unknown	Unknown	Blood	Bouvet & Jeanjean RM 1989, Janssen et al IJSB 1997
LUH 1717 ( = ATCC 17905)		69	21-25-24-15-24-17-24		61	100	0	-	Unknown	Before 1963	Conjunctiva	Bouvet & Jeanjean RM 1989, Janssen et al IJSB 1997
***A.*** ** genomic sp. 15BJ**												
LUH 1729 ( = Adam Ac606)		66	19-23-25-12-22-15-22		62	101	0	-	Unknown	Unknown	Skin	Bouvet & Jeanjean RM 1989, Janssen et al IJSB 1997
LUH 1730 ( = SEIP 23.78)		67	19-23-25-13-22-15-21		62	102	0	-	Unknown	Unknown	Urine	Bouvet & Jeanjean RM 1989, Janssen et al IJSB 1997

nd: not determined.

* Numbers of antimicrobial agents to which an isolate was resistant using disc diffusion with 10 antimicrobial agents.

** Resistance to at least one representative of 3 or more of the 5 classes of antimicrobial agents, i.e. beta-lactams (piperacillin, ceftazidime, ampicillin-sulbactam, imipenem), aminoglycosides (gentamicin, amikacin, tobramycin), fluoroquinolones (ofloxacin), tetracyclines (tertracycline) and the combination of sulfonamide and diaminopyriminide (sulfamethoxazol + trimethoprim).

*** Multiple isolates of outbreak 1–7 were analyzed in the current study to check for reproducibility and concordance (see text); only one strain per outbreak was included in the table.

**** The genome of seven *A. baumannii* strains was fully sequenced (ATCC 17978, AYE, SDF, ACICU, AB0057, AB307-0294 and AB900). For these strains, sequences were extracted from the genome sequence.

***** According to previous publications; criteria may differ from those used for the strains analysed in this work.

Clonal complex 2 (composed of ST2, ST45 and ST47) comprised all clone II strains, with a single exception: strain LUH6049 (ST59) differed from ST2 by three genes and from ST45 and ST47 by two genes. Hence, ST59 cannot be attributed to CC2 by our definition of CCs, which is based on a single allelic mismatch; however, the closest relatives of ST59 are members of CC2. ST45 and ST47 each differed from ST2 by a single gene, *fusA*. CC3 (ST3 and ST14) comprised all strains of clone III, excepted strain LUH5687 (ST13), which differed from ST3 by *fusA* and *recA*.

Additional groups of genetically related but geographically distant isolates were identified, which correspond to the definition of clone *sensu* Orskov and Orskov [36]. CC32 included ST32 together with ST28 and ST53 and included isolates from Denmark and Sweden; three strains of CC32 formed AFLP cluster 6 in the 1996 study by Dijkshoorn *et al*. [7]. CC10 (ST10 and ST23) isolates, previously identified to a tentative novel clone B by AFLP [37], were retrieved in the Czech Republic, the Netherlands and Australia. Finally, ST15 contained nine strains with varied geographic origins (Netherlands, Czech Republic, Argentina). This clone was also identified previously by AFLP analysis and designated tentative clone A [37].

All isolates within a given outbreak set had the same ST (**Table 3**). One outbreak corresponded to ST2 (Rotterdam 1982), whereas two fell in CC1: Venlo 1986 (ST1) and Leiden 2003 (ST20). The four remaining outbreaks were caused by four distinct STs (ST5, ST15, ST16 and ST52). ST52 caused an outbreak in Enschede (The Netherlands) in year 1986 and also included ATCC19096^T^, the type strain of *A. baumannii*, which was isolated before 1949; strains of ST52 were previously included in AFLP cluster C [37].

Strains that have been subjected to genome sequencing were mapped onto the MLST population framework by retrieving their MLST gene sequences. The three strains AB0057 [38], AB307-0294 [38] and AYE [39], [40] fell into ST1, consistent with their genome sequences showing >99.9% similarity at orthologous genes [38]. The multidrug resistant strain ACICU [41] fell in ST2, whereas the susceptible strain AB900 [38] fell into ST49, a double-locus variant of ST3. Finally, strain ATCC 17978 [42] isolated from a 4-month-old infant with fatal meningitis, corresponded to the singleton ST77, while the genome-decaying strain SDF [39], [40] had ST17 (**Fig. 2**).

### Comparison of MLST with AFLP data

AFLP data were obtained for *A. baumannii* strains of this study (**Table 3**). In previous ‘polyphasic’ studies, combining several genotypic and phenotypic methods, a similarity level of ∼80% was deduced as the cut-off level to identify clones among sets of well-defined strains [7], [43]. Fifty-six STs and 48 AFLP types (80% cut-off) were distinguished, resulting in a similar discriminatory power (Simpson's index 91.7 vs. 91.4, respectively; 95% confidence interval 88.9 – 94.4 and 88.8 – 94.0, respectively) using this AFLP cut-off. Comparison of MLST data with AFLP data showed almost complete agreement with respect to assignment to clones (**Table 3**). The two minor exceptions were LUH5687, clone III by AFLP, but being a double-locus variant of ST3, thus not being included in CC3; and LUH6049, a clone II strain by AFLP which showed four allelic mismatches with ST2 (but was still linked to ST2 by the MStree algorithm, **Fig. 2**). Accordingly, a vast majority of strains within CC1, CC2, CC3, CC10 and CC32 had the same AFLP type. Strains with the same ST were always of the same AFLP type, with the only exceptions of ST2 (the most frequent) and ST71 (gen. sp. 13TU). However, AFLP fingerprints in strains of ST2 and ST71 were highly similar, indicating microevolution from a common ancestor, thus being consistent with MLST.

When typing strains in hospital epidemiology, a distinct AFLP cut-off level (90%) is generally used [28]. Using this cut-off, 88 AFLP types were distinguished, resulting in a Simpson's index of 98.5%, and the central STs of the three European clones I, II and III were subdivided into three, 15 and 13 AFLP types, respectively (**Table 3**). Thus, for local epidemiology purposes, AFLP is more discriminatory than MLST.

### Antimicrobial susceptibility of clonal complexes

**Figure 3:**
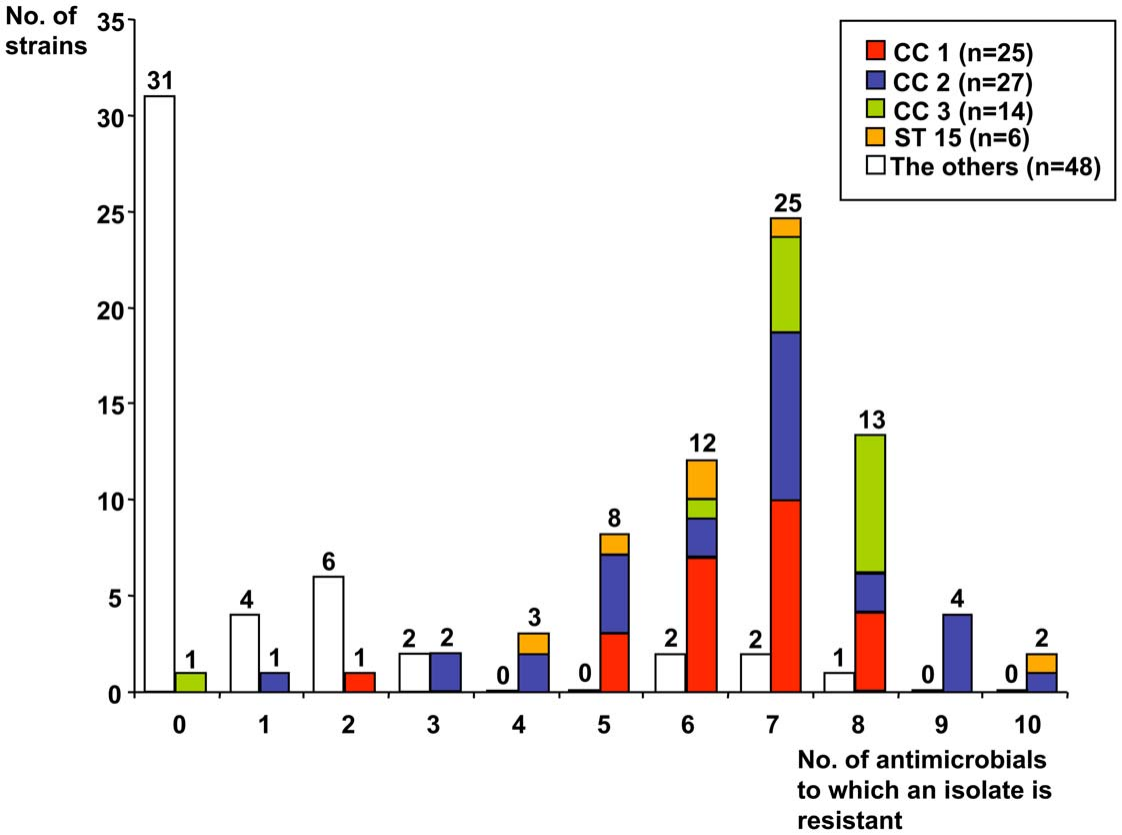
Distribution of *A. baumannii* isolates according to the level of multidrug resistance and their genotype. The isolates allocated to four multidrug resistant international clones (CC1 to CC3 and ST15; colors as on **Fig. 2**) are compared with other *A. baumannii* isolates. Each of the seven outbreak sets (see text) was represented by only one isolate. Note that most members of clones CC1 to CC3 and ST15 are resistant to multiple antimicrobial agents, whereas most isolates of other genotypes are not.

Susceptibility to 10 antimicrobial agents representing five antimicrobial classes was investigated. Multidrug resistance was found only in *A. baumannii* strains. Importantly, MDR strains were distributed into a limited number of STs, which corresponded almost exclusively to international clones including CC1, CC2, CC3 and ST15. Conversely, these clones comprised almost exclusively MDR strains (**Figure 3**): all isolates of CC1, CC2, CC3 and ST15 were MDR except for three isolates (one in each of CC1, CC2 and CC3). MDR strains of CC1, CC2, CC3 and ST15 showed resistance to 5–8, 3–10, 6–8 and 4–10 antimicrobials, respectively. The number of different resistance profiles was 16, 18, 4, and 5, respectively (**Table S1**). Compared to CC1, CC2 and ST15, MDR strains of clone III were relatively homogeneous in their resistance profiles, differing only in susceptibility to ceftazidime and/or piperacillin.

Seven other MDR strains belonged to ST5, ST10, ST16, ST29, ST46, ST50 and ST59. Notably, two of these MDR strains represented outbreak sets included in this study. Out of these seven outbreak sets, all but one (ST52) included MDR strains. MDR strains belonging to ST15, CC10 and ST52 are included in the AFLP clusters A, B and C, respectively, of a previous study [37].

Susceptibility testing to carbapemens showed nine strains that were resistant to imipenem and were also resistant to meropenem (**Table S1**). These carbapenem-resistant strains were found exclusively in CC2 (7 strains) or ST15 (2 strains).

## Discussion

The main purpose of this study was to determine the population structure of *A. baumannii* and to characterize the genetic diversity and distinctness of groups of isolates previously ascribed to international clones. In addition, we determined the extent of phylogenetic distinctness of *A. baumannii* from other species. Our results demonstrate a striking contrast between the low amounts of average nucleotide divergence within *A. baumannii* (0.35%) and the large genetic distance of this species from gen. sp. 13TU (4.65%), its closest relative. This result is consistent with recent findings [25], [35] and fully supports the taxonomic distinction of these two sequence clusters [44].

The average genetic divergence between *A. baumannii* isolates (0.35%) is comparable to e.g. *Klebsiella pneumoniae* (0.37%) [45], but both are atypically homogeneous compared to many bacterial species, including other nosocomial pathogens such as *Escherichia coli* [46], [47]. Whereas strains within typical bacterial species can diverge by up to 5% at orthologous genes [48], no pair of *A. baumannii* strains was found to diverge by more than 0.77% (i.e., roughly 3 nucleotide differences per gene portion on average), even though our isolates were selected to represent the breadth of currently known genetic diversity of *A. baumannii*.

Low amounts of polymorphism may indicate that *A. baumannii* experienced a severe bottleneck (i.e., a reduction of population size) relatively recently, with little time having elapsed since then for diversity to accumulate again. One hypothesis would be that the bottleneck was a consequence of a narrow ecological niche of *A. baumannii*. Indeed, this species seems relatively rare in human carriage and almost never found in soil [2]. Other *Acinetobacter* species have a broader distribution as soil dwellers or as commensals of human skin [2]. Thus, if the ecological niche of *A. baumannii* were more restricted than that of other species, its population size may have been contracted by ecological changes that reduced its habitat. The lack of phylogenetic structure within the species is consistent with the simultaneous diversification of multiple lineages due to rapid population expansion following a bottleneck, resulting in a star-like phylogeny. An alternative possibility would be that clinical isolates of *A. baumannii* do not fully represent the diversity of the species, and instead constitute a restricted subset that acquired the ability to colonize and infect humans. Recent studies reported *A. baumannii* from animals and vegetables [2], [49], [50], and the metabolic versatility of a clone I isolate [40] is indicative of adaptation to diverse habitats. It will be very important to assess the diversity of isolates from non-clinical sources to better understand *A. baumannii* population structure, ecology and epidemiological dynamics.

Identification of species of the *Acb* complex using phenotypic methods is difficult [31], [51], while validated genotypic identification methods, such as amplified 16S ribosomal DNA restriction analysis [52] or AFLP analysis [53] require reference databases for identification and are not widely applied. Sequence-based methods provide clear advantages for identification [35], [54]. Sequencing of a single gene already provides good identification confidence, given that no case of strain misplacement was found in the seven individual gene phylogenies, in contrast to other bacterial groups such as genera *Streptococcus* and *Neisseria* (e.g., [32]). The apparent absence or rarity of sequence replacement may indicate a loss of ability for homologous recombination, even though the genes for natural competence are present in *A. baumannii* [40] and strains of *A. baumannii* clearly are able to incorporate foreign DNA. Alternately, it is possible that an ecological barrier, which would limit opportunity for DNA exchange, has arisen between *Acinetobacter* species following adaptation towards distinct niches. Still, identification based on at least two genes should be considered more reliable, as horizontal gene transfer can theoretically happen and would lead to wrong identification. In addition, multiple genes buffer against the distorting effect of recombination on phylogenies, as was observed for *rplB*.

MLST analysis of *A. baumannii* strains revealed a high degree of discrimination, consistent with previous MLST studies [24], [25]. The selected housekeeping genes were successfully amplified and sequenced in all strains of *A. baumannii*, *A. calcoaceticus*, gen. sp. 3 and gen. sp. 13TU, as well as in the distant gen. sp. 13BJ and 15BJ, suggesting applicability of this MLST scheme to many *Acinetobacter* species. Strain discrimination among strains of species other than *A. baumannii* was also found previously [24], [25].

Clonal groups within bacterial species often differ by their biological properties, such as virulence or epidemicity [21]–[23]. Typically, these groups are identified by determining phylogenetic relationships among MLST genotypes based on allelic profiles (**Fig. 2**), rather than nucleotide sequences (**Fig. S2**), as the former approach is less sensitive to strong distortions caused by homologous recombination [21]. The MStree analysis revealed only five clonal complexes, three of which (CC1 to CC3) corresponded to international clones I to III. For consistency, we baptized as ST1, ST2 and ST3 their central and most prevalent genotypes, which most likely represent the founder of their group [21]. Our results now show formally that clones I – III correspond to typical MLST clonal complexes that can readily be demarcated from other *A. baumannii* genotypes. Thus, MLST data fully confirm the clonal nature of clones I to III, which was initially inferred from several characteristics including PFGE, protein profile, AFLP or ribotyping [7], [8]. In addition, because the genetically central genotype is numerically highly dominant within each CC (with an extreme situation of ST15), our data are suggestive of very fast clonal expansions, with too little time having elapsed to allow genetic differentiation of many variants. These results thus fit with epidemiological knowledge gathered over the two last decades, as countless reports of outbreaks caused by multiresistant isolates of clones I to III reflect their rapid clonal spread.

Recent evidence shows that beyond the three early recognized clones I to III, multiple clones of *A. baumannii* have large geographic distributions [18], [19]. ST15, CC10, ST52 and CC32 can be regarded as novel international clones, and they correspond to previously identified AFLP clusters A, B and C [37] and cluster 6 [7], respectively. Rather than giving roman numerals or letters to novel widespread clones, we would recommend to follow the widely successful MLST-based naming system, which proved convenient for other bacterial species [21], [22], [55], [56]. Clones are simply designated by their ST or CC number, with clonal complexes being numbered after the ST number of their central and/or more prevalent genotype (e.g., CC1 to CC3 for clones I to III, respectively). We propose that MLST characterization should be used as a reference to compare *A. baumannii* strains across studies, as is now the case in nearly 100 bacterial species (mlst.net; pubmlst.org; mlst.ucc.ie; www.pasteur.fr/mlst). For this purpose, a publicly available *A. baumannii* MLST web site was set-up at www.pasteur.fr/mlst.

We estimated the ratio of recombined to mutated nucleotides during the diversification of clonal complexes [57] at 1.3 (four alleles with two changes, attributed to recombination, versus 6 alleles with one change only, attributed to mutation), similar to the recombination/mutation ratio estimated using the bayesian method ClonalFrame (0.96; confidence interval 0.63 – 1.45). These results indicate that *A. baumannii* is not a highly recombining species, even though it should be noted that detection of recombination is difficult due to the very low polymorphism of *A. baumannii*. Therefore, clones defined as widespread STs or CCs are likely to be genetically stable and recognizable over very long periods of time (possibly in the order of thousands or more years), as in other bacterial species with low or moderate homologous recombination rates [58]. It is therefore predictable that isolates can belong to the same ST and be genomically highly similar [38] even though they were isolated decades apart (see members of ST1, ST2 and ST52; **Table 3**). The genetic diversity within the three major international clones is comparable to that observed e.g. for serovar Typhi of *Salmonella enterica*, which age has been estimated at 50,000 years [59] (even if determining the age of bacterial lineages is highly debatable [60], [61]).

There is rapidly growing genome-wide evidence that members of a single clone can differ by the presence or absence of resistance genes, resistance islands and mobile elements [38]–[41], [62]. For example, several structures of the resistance island are distinguished among clone I members [17], [38], [39] and integron structures and resistance gene content can differ among members of same clone from different geographic regions, while the same mobile elements can be transferred horizontally between members of distinct clones [63]. Hence, MLST genotypes can be regarded as evolutionary vessels with a stable core genome, while their accessory genome, including resistance determinants, undergo rapid evolution. As a consequence, finer typing of isolates that belong to widespread clones is highly necessary for epidemiological purposes and to distinguish within clones, subtypes with particular gene content, phenotype and geographic distribution [64].

The evolutionary success of the international clones currently remains unexplained. Among the distinctive characteristics of the international clones, multidrug resistance to antimicrobial agents is clearly the most salient, as noted early [7], [9], [10]. There is a strong dichotomy in the *A. baumannii* population between these clones and other members of the species, which are mostly susceptible and only occasionally cause infection. This emphasizes that not all *A. baumannii* strains are a priori problematic in the hospital setting [28]. Comparisons of features that could favor the widespread clones as colonizers or pathogens in hospitals, such as resistance to desiccation [65] or disinfection [66], biofilm formation [67] or adherence to human cells [68], have so far failed to distinguish isolates belonging to successful clones from other genotypes. Hence, antimicrobial resistance may represent the main reason for the evolutionary success of international clones. Possibly, an increased propensity of these clones to colonize and cause infection in humans exposed them to increased levels of antimicrobials. Alternately, these particular clones may be more prone to acquire foreign genetic material. It will be interesting to determine whether large resistance islands detected in members of clones I and II [17], [38], [39], [41] are a distinctive feature of widespread clones, and whether these clones are carried more frequently by humans, even if at low levels.

It is not yet clear whether the association of multidrug-resistance and clones results primarily from the spread of already established MDR strains, or rather from independent acquisition of resistance determinants by susceptible strains of the same clone. The former scenario can possibly be applied to clone III, which except for one fully susceptible strain, showed nearly identical resistance profiles and genotypes and included recent isolates [63]. In contrast, the situation in clone I, clone II and ST15 is more complex and may result from the fact that these clones are older and thus have undergone many genetic events associated with resistance determinants. Different selection pressures and genetic pools providing resistance determinants, as well as instability of some resistance determinants, all could contribute in explaining the observed intra-clonal diversity.

In conclusion, our study shows that *A. baumannii* populations of clinical isolates have a genetically highly homogeneous core genome. The phylogenetic structure is indicative of two disjoint waves of expansion: the first wave followed a severe bottleneck that occurred at some undetermined time in the distant past, while a second wave is now developing through the rapid expansion of a limited number of multi-resistant clones that become highly problematic as nosocomial infectious agents.

## Materials and Methods

### Bacterial strains

A total of 173 *Acinetobacter* strains were characterized (**Table 3**). Most isolates were from clinical origin and were, with few exceptions, collected between 1987 and 2005, mainly in European countries. First, 123 genotypically distinct and epidemiologically unrelated *A. baumannii* strains (‘diversity set’) were included. These isolates were selected from ∼600 isolates (excluding outbreak replicates) from the Leiden University Medical Center AFLP database, such that the selection displayed the maximal diversity at the 90% AFLP similarity cut-off level, and was also diverse in time-space origin. Previous studies have used the ∼80% AFLP similarity level as a cut-off for defining major clones [43]. Thus, the diversity set included 25 strains of the international (previously named ‘European’) clone I, 30 of clone II, and 15 of clone III (**Table 3**). Second, 24 additional *A. baumannii* isolates from 7 outbreaks for which one representative was included in the diversity set, were investigated for reproducibility and epidemiological concordance. Isolates of each of the seven outbreaks had an AFLP similarity ≥90% and were from the same time-space origin. Apart from these, there were 48 additional *A. baumannii* isolates of the diversity set that were from known outbreaks (**Table 3**). These isolates were considered to represent an outbreak if they shared with other isolates a common time-space origin and a common genotype and/or a common antibiotic susceptibility profile. Isolates were not considered to be part of an outbreak (**Table 3**) if local data (typing and epidemiology) showed no evidence for this. If there was no indication that a strain belonged to an outbreak or not, they were labeled as ‘outbreak unknown’. Third, we included the seven *A. baumannii* strains (ATCC 17978, AYE, SDF, ACICU, AB0057, AB307-0294 and AB900) for which a complete genome sequence was published; the sequences of the gene portions corresponding to the MLST templates were extracted from the genome sequences [38]–[42]. Finally, we included 15 isolates of the species that are closely related to *A. baumannii* (*A. calcoaceticus*, *A.* gen. sp. 3 and 13TU), and four isolates of *Acinetobacter* gen. sp. 13BJ and 15BJ (used as outgroups for the phylogenetic analysis).

### AFLP

AFLP data were generated as described [28]. DNA was digested with *Eco*RI and *Mse*I simultaneously with adapter ligation. PCR was done with a Cy5-labelled *Eco*RI+A primer and a *Mse*I*+*C primer (A and C, selective nucleotides). Amplified fragments were separated with the ALF II express system (Amersham Biosciences, Roosendaal, The Netherlands). Digitized fingerprints were analyzed using Pearson's coefficient as a similarity measure and unweighted pair group method with arithmetic mean (UPGMA) linkage for clustering with BioNumerics software 4.1 (Applied-Maths, St-Martens-Latem, Belgium).

### Antimicrobial susceptibility testing

Susceptibility was tested by disc diffusion following the CLSI recommendations using Mueller–Hinton agar (Oxoid, Basingstoke, UK) and 10 antimicrobial agents, which are primarily effective against *A. baumannii* [37]. The resistance breakpoints were adjusted according to the known distribution of inhibition zone diameters among *A. baumannii* strains. These values were identical to those of the CLSI for intermediate susceptibilities except for tetracycline and piperacillin, for which the CLSI values for resistance were used. The agents (µg per disc; resistance breakpoint in mm) included ampicillin+sulbactam (10+10; ≤14), piperacillin (100; ≤17), ceftazidime (30; ≤17), imipenem (10; ≤15), gentamicin (10; ≤14), tobramycin (10; ≤14), amikacin (30; ≤16), ofloxacin (5; ≤15), sulfamethoxazole+trimethoprim (23.75+1.25; ≤15) and tetracycline (30; ≤14) (Oxoid). Multidrug resistance was defined as resistance to at least one representative of three or more of the five classes of antimicrobial agents, i.e. beta-lactams, aminoglycosides, fluoroquinolones, tetracyclines or the combination of sulfonamide and diaminopyrimidine.

### Multilocus Sequence Typing (MLST)

Primer pairs were designed for PCR amplification and sequencing of internal portions of seven housekeeping genes (**Table 4**). Three of these pairs (*cpn60*, *gltA* and *recA*) were designed by Bartual *et al.* [24]. Primer pairs for three other genes, which are present in most bacterial phyla (*fusA*, *pyrG* and *rplB*), were designed by adapting, using the *A. baylyi* and *A. baumannii* genome sequences, the primers initially proposed by Santos and Ochman [69]. Finally, primers for gene *rpoB* were designed previously [70]. The portion of *rpoB* that was amplified with these primers corresponds to positions 1,681 to 2,136. These genes represent seven distinct loci on the *A. baumannii* chromosome (**Table 4**). The internal gene portions chosen for MLST allele and profile definition ranged in length from 297 bp (*pyrG*) to 633 bp (*fusA*). Further details on this MLST scheme can be found at www.pasteur.fr/mlst. Nucleotide sequences were obtained using Big Dye version 1.1 chemistry on an ABI 3730XL apparatus.

**Table 4:** Primers used for MLST.

Locus	Putative function of gene	Forward primer	Reverse primer	Location (a)
*cpn60 (b)*	60-KDa chaperonin	5′- ACTGTACTTGCTCAAGC -3′	5′- TTCAGCGATGATAAGAAGTGG -3′	3,089,652–3,089,248
*fusA*	protein elongation factor EF-G	5′- ATCGGTATTTCTGCKCACATYGAT -3′	5′- CCAACATACKYTGWACACCTTTGTT -3′	1,008,107–1,008,739
*gltA (b)*	citrate synthase	5′- AATTTACAGTGGCACATTAGGTCCC -3′	5′- GCAGAGATACCAGCAGAGATACACG -3′	3,143,730–3,143,248
*pyrG*	CTP synthase	5′- GGTGTTGTTTCATCACTAGGWAAAGG -3′	5′- ATAAATGGTAAAGAYTCGATRTCACCMA -3′	2,201,622–2,201,326
*recA (b)*	homologous recombination factor	5′- CCTGAATCTTCYGGTAAAAC -3′	5′- GTTTCTGGGCTGCCAAACATTAC -3′	2,274,422–2,274,793
*rplB*	50S ribosomal protein L2	5′- GTAGAGCGTATTGAATACGATCCTAACC -3′	5′- CACCACCACCRTGYGGGTGATC -3′	3,557,351–3,557,022
*rpoB*	RNA polymerase subunit B	5′- GGCGAAATGGCDGARAACCAC -3′	5′- GARTCYTCGAAGTTGTAACC -3′	307,298–307,753

(a) On *Acinetobacter baumannii* ATCC17978, complete genome (NC009085).

(b) Primers from Bartual et al., 2003.

### Data analysis

Sequence chromatograms were edited and stored using BioNumerics v5.10. To achieve high levels of confidence on each nucleotide substitution, all nucleotides within the internal gene portion chosen for MLST analysis were supported by at least two sequence chromatograms. For a given locus, a novel allele number was attributed to each distinct sequence, and a distinct sequence type (ST) number was attributed to each distinct combination of alleles at the seven genes. Allele sequences and allelic profiles are available on Institut Pasteur's MLST web site at www.pasteur.fr/mlst. Relatedness between the different STs was investigated based on comparison of allelic profiles using the minimum spanning tree (MStree) method from BioNumerics. We used the classical criterion of one allelic mismatch to group STs into clonal complexes [21]. Nucleotide diversity was calculated using DNAsp v4 [71]. MEGA [72] was used to compute and draw phylogenetic trees using the Jukes and Cantor substitution model. Simpson's index was calculated using the web resource www.comparingpartitions.info. ClonalFrame analysis was performed following the developer's instructions [73].

## Supporting Information

Figure S1 Individual gene phylogenies. Phylogenetic analysis of 173 Acinetobacter strains of several named and unnamed species, based on seven individual genes using the neighbor-joining method with Jukes-Cantor distance. Symbols as on Figure 1.
(0.11 MB PDF)

Figure S2 Intra-specific phylogenetic structure of A. baumannii. An unrooted neighbor-joining phylogenetic analysis of concatenated sequences of the seven MLST genes was performed. Numbers at the tip of the branches correspond to the sequence type (ST) number. Clones I to III (CC1 to CC3) are circled.
(0.08 MB PPT)

Table S1 Antimicrobial susceptibility of A. baumannii isolates.
(0.01 MB PDF)
