# Supplementary figures and images for: The Population Structure of Acinetobacter baumannii: Expanding Multiresistant Clones from an Ancestral Susceptible Genetic Pool
Source: PLoS One. 2010 Apr 7;5(4):e10034. doi: 10.1371/journal.pone.0010034 (PMC2850921; doi:10.1371/journal.pone.0010034)

Figure S1

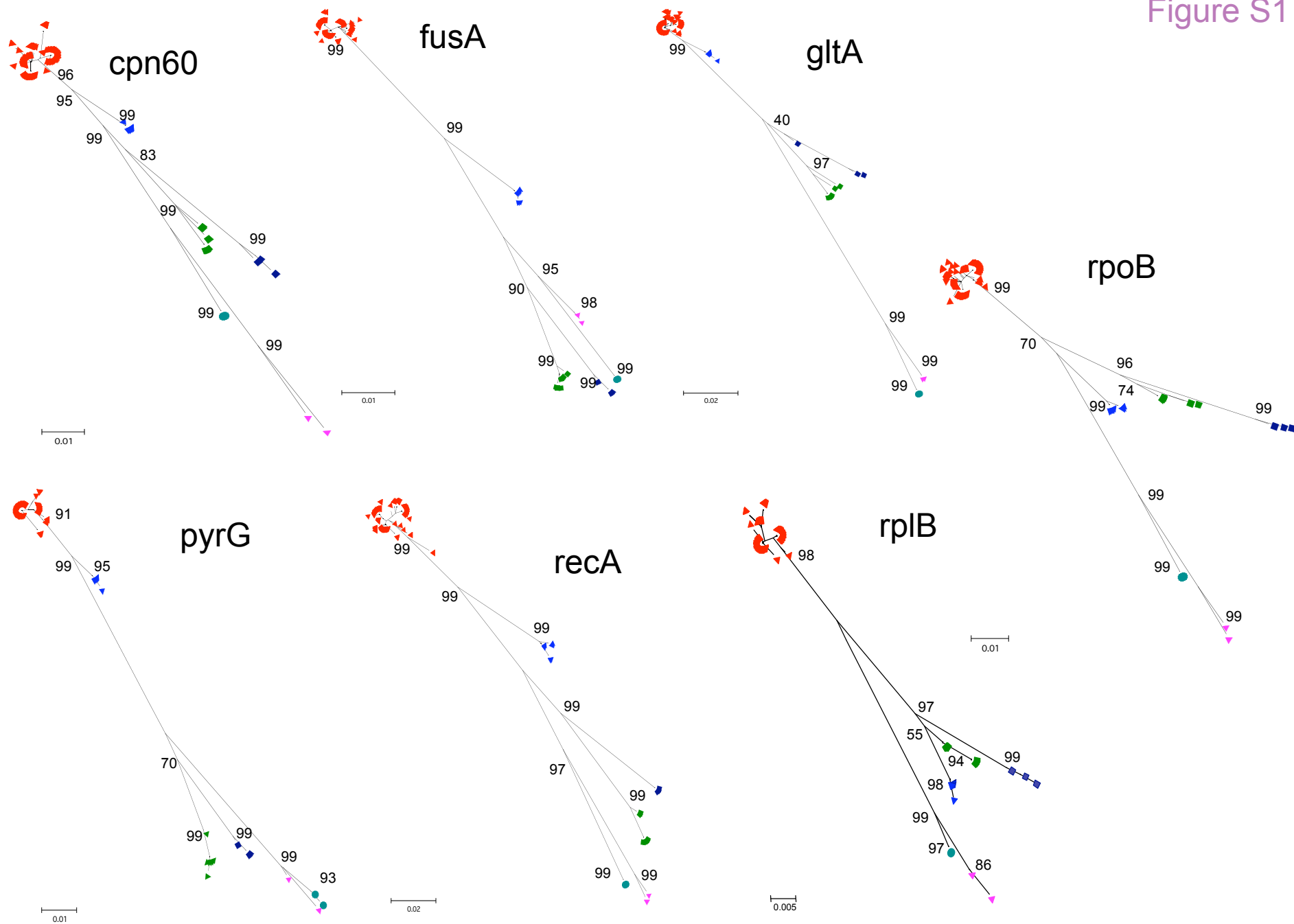

Supplement: Figure S1 — Individual gene phylogenies. Phylogenetic analysis of 173 Acinetobacter strains of several named and unnamed species, based on seven individual genes using the neighbor-joining method with Jukes-Cantor distance. Symbols as on Figure 1. (0.11 MB PDF) [file pone.0010034.s001.pdf]

## Slide 1
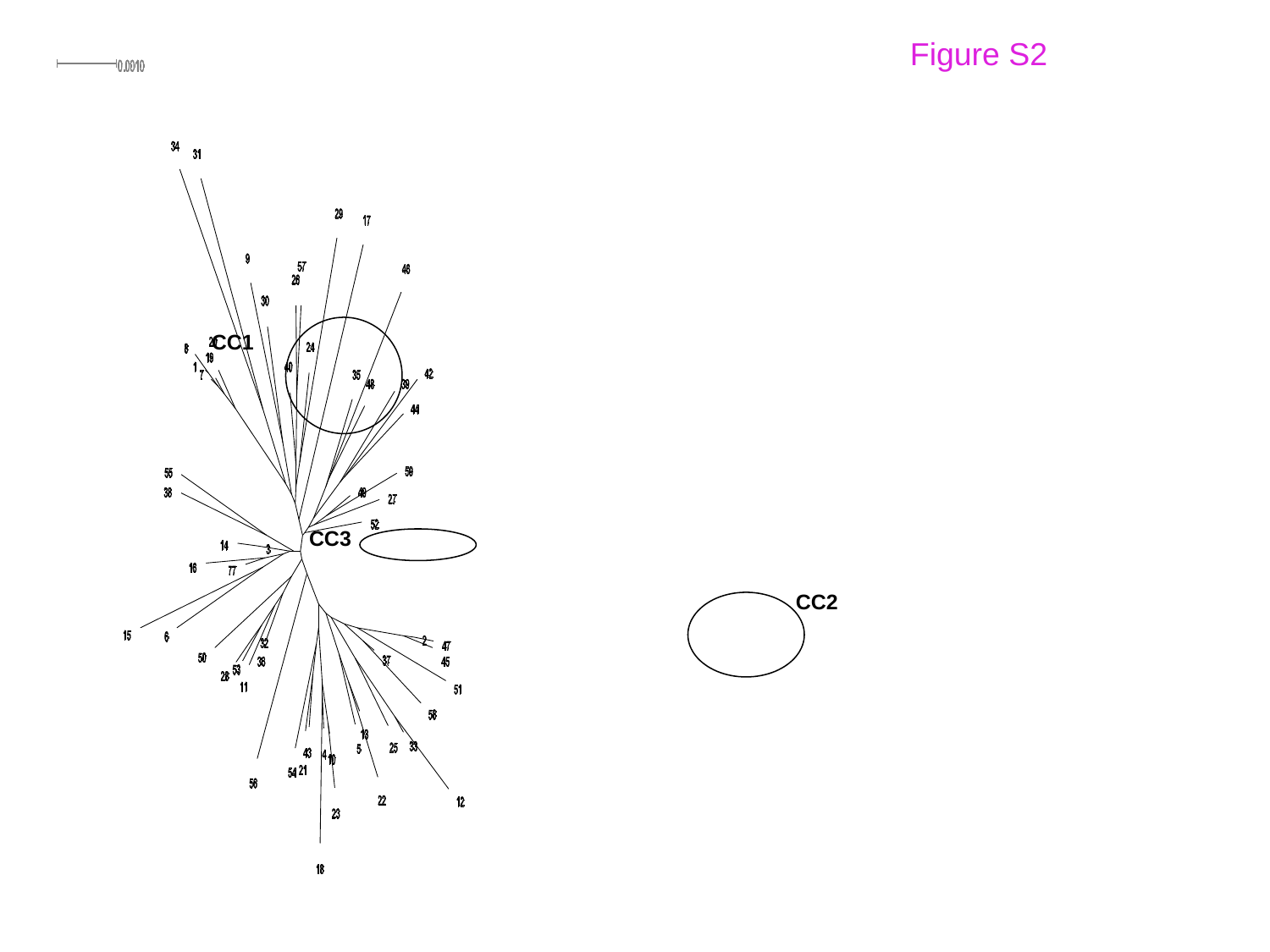

Figure S2
CC1
CC3
CC2

Supplement: Figure S2 — Intra-specific phylogenetic structure of A. baumannii. An unrooted neighbor-joining phylogenetic analysis of concatenated sequences of the seven MLST genes was performed. Numbers at the tip of the branches correspond to the sequence type (ST) number. Clones I to III (CC1 to CC3) are circled. (0.08 MB PPT) [file pone.0010034.s002.ppt]
