# Supplementary material for: The Population Structure of Acinetobacter baumannii: Expanding Multiresistant Clones from an Ancestral Susceptible Genetic Pool
Source: PLoS One. 2010 Apr 7;5(4):e10034. doi: 10.1371/journal.pone.0010034 (PMC2850921; doi:10.1371/journal.pone.0010034)
Supplement: Table S1 — Antimicrobial susceptibility of A. baumannii isolates. (0.01 MB PDF) [file pone.0010034.s003.pdf]

Table S1. Antimicrobial susceptibilities of 120 *A. baumannii* isolates.

Strain no.	ST	City, country, year of isolation	No. of resistan-cies	Resistance (R) or susceptibility (S) to									
				Piperacillin	Ceftazidime	Ampicillin + sulbactam	Imipenem	Gentamicin	Amikacin	Tobramycin	Ofloxacin	Tetracycline	Cotrimoxazole
LUH 3783 (= NIPH 10)	1	Prague, CZ, 1991	8	R	R	R	S	R	R	S	R	R	R
LUH 4576 (= NIPH 56)	1	Prague, CZ, 1992	2	S	S	S	S	S	S	S	S	R	R
LUH 4624 (= NIPH 470)	1	C. Budejovice, CZ, 1997	6	R	R	S	S	R	R	S	R	R	S
LUH 4640 (= NIPH 321)	1	Tábor, CZ, 1994	7	R	S	R	S	R	R	S	R	R	R
LUH 6015 (= 11A352)	1	Rome, IT, 1998	5	S	R	S	S	R	S	S	R	R	R
LUH 6224	1	Sydney, AU, 1995	8	R	S	R	S	R	R	R	R	R	R
LUH 7140 (= A789)	1	London, UK, 2000	6	R	R	S	S	R	S	S	R	R	R
LUH 8592	1	Sofia, BG, 2001	7	R	R	S	S	R	R	S	R	R	R
LUH 9668	1	Dublin, IE, 2003	6	R	S	R	S	R	S	S	R	R	R
RUH 0875	1	Dordrecht, NL, 1984	6	R	S	R	S	R	S	R	S	R	R
RUH 2037	1	Venlo, NL, 1986	7	R	R	R	S	R	S	S	R	R	R
RUH 3238 (= GNU 1084)	1	Sheffield, UK, 1987	5	R	S	R	S	R	S	S	S	R	R
RUH 3239 (= GNU 1083)	1	London, UK, 1985-88	6	R	R	R	S	R	S	S	S	R	R
RUH 3242 (= GNU 1082)	1	Basildon, UK, 1989	6	R	R	R	S	R	S	S	S	R	R
RUH 3247 (= GNU 1078)	1	Leuven, BE, 1990	7	R	S	R	S	R	S	R	R	R	R
RUH 3282 (= GNU 1079)	1	Salford, UK, 1990	8	R	R	R	S	R	R	S	R	R	R
LUH 6014 (= 11A221)	1	Rome, IT, 1998	7	R	R	S	S	R	R	S	R	R	R
LUH 6050 (= 36C058)	1	Pretoria, ZA	7	R	R	S	S	R	S	R	R	R	R
LUH 6013 (= 11A018)	1	Rome, IT, 1997	6	S	R	S	S	R	R	S	R	R	R
LUH 5881 (= 17C078)	1	Madrid, ES, 1998	7	R	R	S	S	R	R	R	R	S	R
LUH 6125 (= 14C052)	1	Krakow, PL, 1998	7	R	R	S	S	R	R	S	R	R	R
LUH 3788 (= NIPH 24)	2	Prague, CZ, 1991	6	R	S	R	S	R	R	S	S	R	R
LUH 4629 (= NIPH 657)	2	Prague, CZ, 1996	7	R	R	S	S	R	R	S	R	R	R
LUH 5682	2	Utrecht, NL, 1993	5	R	S	R	S	R	S	S	S	R	R
LUH 6024 (= 16A502)	2	Sevilla, ES, 1998	7	R	R	S	S	R	R	S	R	R	R
LUH 8065	2	Amsterdam, NL, 2001	8	R	R	S	R	R	S	R	R	R	R
LUH 8488	2	Leeuwarden, NL, 2003	7	R	R	S	S	R	R	R	R	R	S
LUH 9233 (= NIPH 1945)	2	Prague, CZ, 2003	7	R	R	R	S	R	S	S	R	R	R
RUH 0134	2	Rotterdam, NL, 1982	5	R	S	R	S	R	S	S	S	R	R
RUH 3240 (= GNU 1086)	2	Newcastle, UK, 1989	4	R	S	S	S	R	S	S	S	R	R
RUH 3245 (= GNU 1080)	2	Salisbury, UK, 1989	3	S	S	S	S	R	S	S	S	R	R
RUH 3422 (= PGS 189)	2	Odense, DK, 1984	1	S	S	S	S	S	S	S	S	R	S
LUH 6025 (= 16A528)	2	Sevilla, ES, 1998	7	R	R	S	S	R	R	S	R	R	R
LUH 6045 (= 18C144)	2	Barcelona, ES, 1997	9	R	R	R	R	R	S	R	R	R	R
LUH 6051 (= 36D042)	2	Pretoria, ZA	4	R	R	S	S	R	S	S	S	R	S
LUH 5868 (= 06A102)	2	Lille, FR, 1997	9	R	R	R	S	R	R	R	R	R	R
LUH 6021 (= 14C003)	2	Krakow, PL, 1998	8	R	R	R	S	R	R	S	R	R	R
LUH 7154 (= A1850)	2	Berkshire, UK, 2000	5	R	R	S	S	S	S	S	R	R	R
LUH 8143	2	Singapore, SG, 1997	7	R	R	R	R	S	S	S	R	R	R
LUH 8533	2	London, UK,	7	R	R	R	S	R	S	S	R	R	R
RUH 3381 (= GNU 666)	2	Cork, IE, 1989	3	R	S	S	S	R	S	S	S	S	R
LUH 5089	2	Warsaw, PL, before 1999	6	R	R	S	S	S	R	S	R	R	R
LUH 6231	2	Sydney, AU, 1999	9	R	R	R	R	R	S	R	R	R	R
LUH 6038 (= 18A350)	2	Barcelona, ES, 1998	9	R	R	R	R	R	R	S	R	R	R
LUH 6034 (= 17C003)	2	Madrid, ES, 1997	10	R	R	R	R	R	R	R	R	R	R
LUH 6126 (= 15A250)	2	Coimbra, PT, 1998	7	R	R	R	S	R	S	S	R	R	R
LUH 5875 (= 12A133)	3	Utrecht, NL, 1997	8	R	R	S	S	R	R	R	R	R	R
LUH 6009 (= 04C048)	3	Paris, FR, 1997	8	R	R	S	S	R	R	R	R	R	R

Table S1. Antimicrobial susceptibilities of 120 *A. baumannii* isolates (*continued: page 2/3*).

Strain no.	ST	City, country, year of isolation	No. of resistan-cies	Resistance (R) or susceptibility (S) to									
				Piperacillin	Ceftazidime	Ampicillin + sulbactam	Imipenem	Gentamicin	Amikacin	Tobramycin	Ofloxacin	Tetracycline	Cotrimoxazole
LUH 6012 (= 10C070)	3	Genoa, IT, 1998	8	R	R	S	S	R	R	R	R	R	R
LUH 6028 (= 16D025)	3	Sevilla, ES, 1998	7	R	S	S	S	R	R	R	R	R	R
LUH 6035 (= 17C085)	3	Madrid, ES, 1998	8	R	R	S	S	R	R	R	R	R	R
LUH 6037 (= 18A155)	3	Barcelona, ES, 1997	8	R	R	S	S	R	R	R	R	R	R
LUH 6215	3	Heerlen, NL, 2000	8	R	R	S	S	R	R	R	R	R	R
LUH 8056	3	Groningen, NL, 2000	8	R	R	S	S	R	R	R	R	R	R
LUH 9536	3	Gent, BE, 1993	7	R	S	S	S	R	R	R	R	R	R
LUH 6020 (= 12A126)	3	Utrecht, NL, 1997	7	R	R	S	S	R	S	R	R	R	R
LUH 6030 (= 16D083)	3	Sevilla, ES, 1997	7	R	S	S	S	R	R	R	R	R	R
LUH 6036 (= 18A025)	3	Barcelona, ES, 1997	7	R	R	S	S	R	S	R	R	R	R
LUH 6048 (= 18D047)	3	Barcelona, ES, 1997	0	S	S	S	S	S	S	S	S	S	S
LUH 8225	4	Leiden, NL, 2002	0	S	S	S	S	S	S	S	S	S	S
LUH 5703	5	Warsaw, PL, 1999	7	R	R	S	S	R	R	R	S	R	R
LUH 3782 (= NIPH 7)	7	Prague, CZ, 1991	7	R	S	R	S	R	R	S	R	R	R
RUH 0510	8	Nijmegen, NL, 1984	5	R	S	R	S	R	S	S	S	R	R
LUH 4633 (= NIPH 190)	9	Prague, CZ, 1993	0	S	S	S	S	S	S	S	S	S	S
LUH 4641 (= NIPH 335)	10	Tábor, CZ, 1994	6	R	S	S	S	R	S	R	R	R	R
LUH 6237	10	Darwin, AU, 1981-91	1	S	S	S	S	S	S	S	S	S	R
LUH 4718 (= NIPH 329)	11	Tábor, CZ, 1994	0	S	S	S	S	S	S	S	S	S	S
LUH 4727 (= NIPH 615)	12	Prague, CZ, 1994	0	S	S	S	S	S	S	S	S	S	S
LUH 5687	13	Utrecht, NL, 1996	0	S	S	S	S	S	S	S	S	S	S
LUH 5874 (= 06A201)	14	Lille, FR, 1997	6	S	S	S	S	R	R	R	R	R	R
LUH 6374	15	Leiden, NL, 2000	5	R	R	S	S	R	S	S	R	R	S
LUH 8102	15	Tilburg, NL, 2000	6	R	S	S	S	R	S	R	R	R	R
LUH 8147	15	Buenos Aires, AR, 1995	4	R	S	S	S	S	R	S	R	S	R
LUH 8406 (= NIPH 1734)	15	M. Boleslav, CZ, 2001	7	R	S	S	R	R	R	R	R	R	S
LUH 8850	15	Leiden, NL, 2003	6	R	S	S	S	R	S	R	R	R	R
LUH 9716	15	Ede, NL, 2004	10	R	R	R	R	R	R	R	R	R	R
LUH 6639	16	Leiden, NL, 2001	8	R	R	R	S	R	R	S	R	R	R
LUH 8326	18	Leiden, NL, 2002	0	S	S	S	S	S	S	S	S	S	S
LUH 8605	19	Sofia, BG, 2002	7	R	R	S	S	R	S	R	R	R	R
LUH 8723	20	Leiden, NL, 2003	8	R	R	S	S	R	R	R	R	R	R
LUH 9415	21	Leiden, NL, 2004	0	S	S	S	S	S	S	S	S	S	S
RUH 1093	22	Rotterdam, NL, 1985	2	S	S	S	S	R	S	S	S	R	S
RUH 1316	23	Rotterdam, NL, 1964	0	S	S	S	S	S	S	S	S	S	S
RUH 1317	24	Rotterdam, NL, 1965	0	S	S	S	S	S	S	S	S	S	S
RUH 1486	25	Rotterdam, NL, 1985	0	S	S	S	S	S	S	S	S	S	S
RUH 1907	26	Rotterdam, NL, 1986	0	S	S	S	S	S	S	S	S	S	S
RUH 2180	27	Nijmegen, NL, 1987	0	S	S	S	S	S	S	S	S	S	S
RUH 2208	28	Malmö, SE, 1980-81	0	S	S	S	S	S	S	S	S	S	S
RUH 3428	28	Malmö, SE, 1980-81	0	S	S	S	S	S	S	S	S	S	S
RUH 3413	29	London, UK, 1981	3	R	S	S	S	S	S	S	S	R	R
RUH 3423	30	Naestved, DK, 1990	0	S	S	S	S	S	S	S	S	S	S
RUH 3424	31	Veile, DK, 1990	2	S	S	S	S	S	S	S	S	R	R
RUH 3425	32	Veile, DK, 1990	0	S	S	S	S	S	S	S	S	S	S
RUH 3429	33	Malmö, SE, 1980-81	1	S	S	S	S	S	S	S	S	R	S
LUH 4631 (= NIPH 60)	34	Prague, CZ, 1992	0	S	S	S	S	S	S	S	S	S	S
LUH 4707 (= NIPH 67)	35	Prague, CZ, 1992	0	S	S	S	S	S	S	S	S	S	S

Table S1. Antimicrobial susceptibilities of 120 *A. baumannii* isolates (*continued: page 3/3*).

Strain no.	ST	City, country, year of isolation	No. of resistan-cies	Resistance (R) or susceptibility (S) to									
				Piperacillin	Ceftazidime	Ampicillin + sulbactam	Imipenem	Gentamicin	Amikacin	Tobramycin	Ofloxacin	Tetracycline	Cotrimoxazole
LUH 4708 (= NIPH 70)	36	Prague, CZ, 1992	0	S	S	S	S	S	S	S	S	S	S
LUH 4709 (= NIPH 80)	37	Prague, CZ, 1993	1	S	S	S	S	S	S	S	S	S	R
LUH 4711 (= NIPH 201)	38	Liberec, CZ, 1992	0	S	S	S	S	S	S	S	S	S	S
LUH 4722 (= NIPH 410)	39	Brno, CZ, 1996	0	S	S	S	S	S	S	S	S	S	S
LUH 4725 (= NIPH 601)	40	Prague, CZ, 1993	0	S	S	S	S	S	S	S	S	S	S
LUH 5684	42	Utrecht, NL, 1994	2	S	S	S	S	R	S	R	S	S	S
LUH 5685	43	Utrecht, NL, 1994	0	S	S	S	S	S	S	S	S	S	S
LUH 5691	44	Utrecht, NL, 1997	0	S	S	S	S	S	S	S	S	S	S
LUH 6011 (= 09A242)	45	Athens, GR, 1997	7	R	R	S	S	R	S	R	R	R	R
LUH 7852 (= NIPH 301)	46	Slaný, CZ, 1994	7	R	R	S	S	R	S	R	R	R	R
LUH 7855 (= NIPH 1362)	47	Prague, CZ, 2000	5	R	R	S	R	S	S	S	R	R	S
LUH 8088	48	Leiden, NL, 2002	0	S	S	S	S	S	S	S	S	S	S
LUH 9084	49	Leiden, NL, 2003	0	S	S	S	S	S	S	S	S	S	S
LUH 9136	50	Leiden, NL, 2004	3	R	S	S	S	S	S	S	S	R	R
RUH 0414	51	Leiden, NL, 1978	0	S	S	S	S	S	S	S	S	S	S
RUH 1063 (= NCTC 7844)	52	*Before 1948*	2	R	S	S	S	S	S	S	S	S	R
RUH 1752	52	Enschede, NL, 1986	0	S	S	S	S	S	S	S	S	S	S
RUH 3023T (= ATCC19606T)	52	*Before 1949*	2	S	S	S	S	R	S	S	S	S	R
RUH 2207	53	Malmö, SE, 1980-81	0	S	S	S	S	S	S	S	S	S	S
RUH 2209 (= ATCC 17904)	54	Before 1962	1	S	S	S	S	S	S	S	S	S	R
RUH 2688	55	Rotterdam, NL, 1987	2	S	S	S	S	R	S	R	S	S	S
RUH 3410	56	London, UK, 1982	0	S	S	S	S	S	S	S	S	S	S
RUH 3414	57	London, UK, 1988	0	S	S	S	S	S	S	S	S	S	S
LUH 6049	59	Ankara, TR, 1997	6	R	R	R	S	R	S	S	S	R	R
